# Advancements in LAM-based diagnostic kit for tuberculosis detection: enhancing TB diagnosis in HIV-negative individuals

**DOI:** 10.3389/fmicb.2024.1367092

**Published:** 2024-02-20

**Authors:** Man Gao, Qianhong Wu, Xinhong Wang, Xiuli Sun, Meng Li, Guanghong Bai

**Affiliations:** ^1^Department of Clinical Laboratory, Shaanxi Provincial Tuberculosis Prevention and Control Hospital, Xi'an, China; ^2^Department of Tuberculosis, Shaanxi Provincial Tuberculosis Prevention and Control Hospital, Xi'an, China; ^3^Department of Surgery, Shaanxi Provincial Tuberculosis Prevention and Control Hospital, Xi'an, China

**Keywords:** pulmonary tuberculosis, extrapulmonary tuberculosis, lipoarabinomannan, chemiluminescence, diagnostic value

## Abstract

**Objective:**

The purpose of this study was to investigate the diagnostic value of urine lipoarabinomannan (LAM) detection based on chemiluminescence assay for pulmonary tuberculosis (PTB) and extrapulmonary tuberculosis (EPTB) in HIV-negative individuals.

**Methods:**

A total of 215 patients and 37 healthy individuals were included according to inclusion and exclusion criteria, including 173 cases of PTB and 42 cases of EPTB. Sputum smears, sputum culture, TB-RNA, GeneXpert, and urine LAM results were obtained from all patients before treatment. Using the composite reference standard as the reference, the diagnostic performance of these methods for PTB and EPTB was evaluated, and the diagnostic performance and cost-effectiveness of different combinations were analyzed.

**Results:**

In PTB, LAM exhibited the highest sensitivity (55.49%), followed by GeneXpert (44.51%). In EPTB, LAM also had the highest sensitivity (40.48%), followed by GeneXpert (33.33%). When combined with one method, LAM combined with GeneXpert showed the highest sensitivity for both PTB (68.79%) and EPTB (61.9%). When combined with two methods, culture, GeneXpert, and LAM showed the highest sensitivity for both PTB (73.99%) and EPTB (69.05%). In terms of cost-effectiveness analysis, the price of LAM was significantly lower than that of GeneXpert ($129.82 vs. $275.79 in PTB and 275.79 vs. 502.33 in EPTB). Among all combinations, the combination of LAM and sputum smear had the lowest cost, with prices of $124.94 for PTB and $263.72 for EPTB.

**Conclusion:**

Urine LAM detection based on chemiluminescence assay can be used as an adjunct diagnostic tool for PTB and EPTB in HIV-negative individuals. This facilitates expanding the current application of urine LAM from solely HIV-positive populations to the general population. LAM detection can overcome the limitations of obtaining clinical samples, and its ease of sample acquisition will be beneficial for its broader application in a larger scope. For economically better-off areas, we recommend using a combination of LAM + GeneXpert+culture for higher sensitivity; for economically disadvantaged areas, LAM + smear microscopy combination can provide a quick and accurate diagnosis of tuberculosis at a lower cost.

## 1 Introduction

Tuberculosis is a disease that plagues all of humanity. According to the Global Tuberculosis Report 2023, there were a total of 10.6 million tuberculosis infections worldwide, an incidence rate of 133 per 100,000 population, and 1.3 million deaths. Tuberculosis is the second largest single infectious cause of death globally, second only to COVID-19 (World Health Organization, 2023).

Traditional diagnostic methods for tuberculosis are time-consuming (sputum culture) or insensitive (sputum smear) (Nicol et al., 2021). Molecular diagnostics, like GeneXpert and TB-RNA, have improved sensitivity, but they have high laboratory requirements and cost, limiting their use in low-income countries (Pang et al., 2014; Zou et al., 2023). These methods primarily rely on the detection of *Mycobacterium tuberculosis* (MTB) in sputum or tissue fluid, which poses significant limitations for patients who do not produce sufficient sputum or have minimal sputum, particularly in the case of pediatric patients (Grant et al., 2012) or severe cases. Additionally, invasive procedures for obtaining tissue fluid samples can cause discomfort to patients. There is a clinical need for a rapid, non-invasive, easily sampled, and highly sensitive and specific diagnostic product for tuberculosis.

Lipoarabinomannan (LAM) is a major component of the cell wall of MTB, accounting for approximately 15% of the bacterial mass (Correia-Neves et al., 2019). LAM is heat and protease stable, and possesses unique structural epitopes specific to MTB (Sigal et al., 2018). LAM can modulate host immunity and plays an important role in the pathogenesis of tuberculosis (Briken et al., 2004). Following MTB degradation, the remaining LAM in the blood is filtered through the glomerular basement membrane into the urine (Bulterys et al., 2019). LAM testing is promising for tuberculosis diagnosis, especially in HIV-positive and disseminated tuberculosis patients (Lawn, 2012). It is a non-invasive test that does not require sputum, and the samples are easy to obtain (Gupta-Wright et al., 2016).

Currently, the Alere Determine TB LAM test is the only commercially available LAM test. It is only recommended by the World Health Organization for the diagnosis of active tuberculosis in HIV-positive individuals (World Health Organization, 2019). However, the sensitivity of AlereLAM varies across different patient groups. Specifically, it is only 16% in patients with a CD4+ T cell count greater than 200 cells/μl, but increases to 45% in those with CD4 counts less than 200 cells/μl (Bjerrum et al., 2020). Within the HIV-positive population, the sensitivity rate of AlereLAM is 42% (Bjerrum et al., 2020), whereas in the HIV-negative population, it is only 18% (Minion et al., 2011).

The HIV attacks the human immune system, ultimately leading to immune deficiency. As a result, MTB can replicate extensively within the body, leading to an increased circulation of LAM. In addition to promoting the replication of MTB, immune suppression also reduces the formation of antigen–antibody complexes, resulting in more free LAM present in the circulation (Lawn, 2012). In late-stage HIV patients, podocyte function is impaired, which leads to an increase in filtered LAM (Minion et al., 2011). Therefore, in patients with co-infection of tuberculosis and HIV, the concentration of LAM in the urine is much higher. In HIV-negative patients, most individuals have normal immune function, which inhibits the replication of MTB and promotes the formation of antigen–antibody complexes. This results in lower concentrations of LAM in the urine. AlereLAM uses immunochromatographic assay based on colloidal gold, with a detection limit of 500 pg./mL (García et al., 2019). This may partially explain why sensitivity of AlereLAM is low, especially in HIV-negative population. FujiLAM uses high-affinity monoclonal antibodies and silver-based amplification, which lowered the detection limit of LAM to 30 pg./mL (Broger et al., 2019). The sensitivity of FujiLAM in the HIV-positive population has reached 70.7% (Broger et al., 2020). However its stability is not satisfactory (Huerga et al., 2023), the utilization of this test kit has not been widespread.

The majority of tuberculosis-infected cases are HIV-negative, resulting in a greater demand for a more stable and sensitive LAM test kit. Chemiluminescence does not require an external light source for excitation, which can reduce background interference and enhance the sensitivity of diagnostics (Roda and Guardigli, 2012). Due to the high technical barriers, there have been limited developments and market promotions of LAM test kits based on the chemiluminescent platform. The reagent kit used in this study is the only commercially available LAM test kit developed on a chemiluminescent platform worldwide (Peng et al., 2023), significantly enhances the sensitivity and stability of LAM detection through urine concentration and chemiluminescent detection method. The limit of detection has been improved to reach 1 pg./mL (according to the user manual provided by the manufacturer).

The purpose of this study is to compare the accuracy of the chemiluminescent-based LAM with other methods for diagnosing tuberculosis, conduct an economic cost-effectiveness analysis of LAM in diagnosing tuberculosis.

## 2 Methods

### 2.1 Study design and population

**Figure 1:**
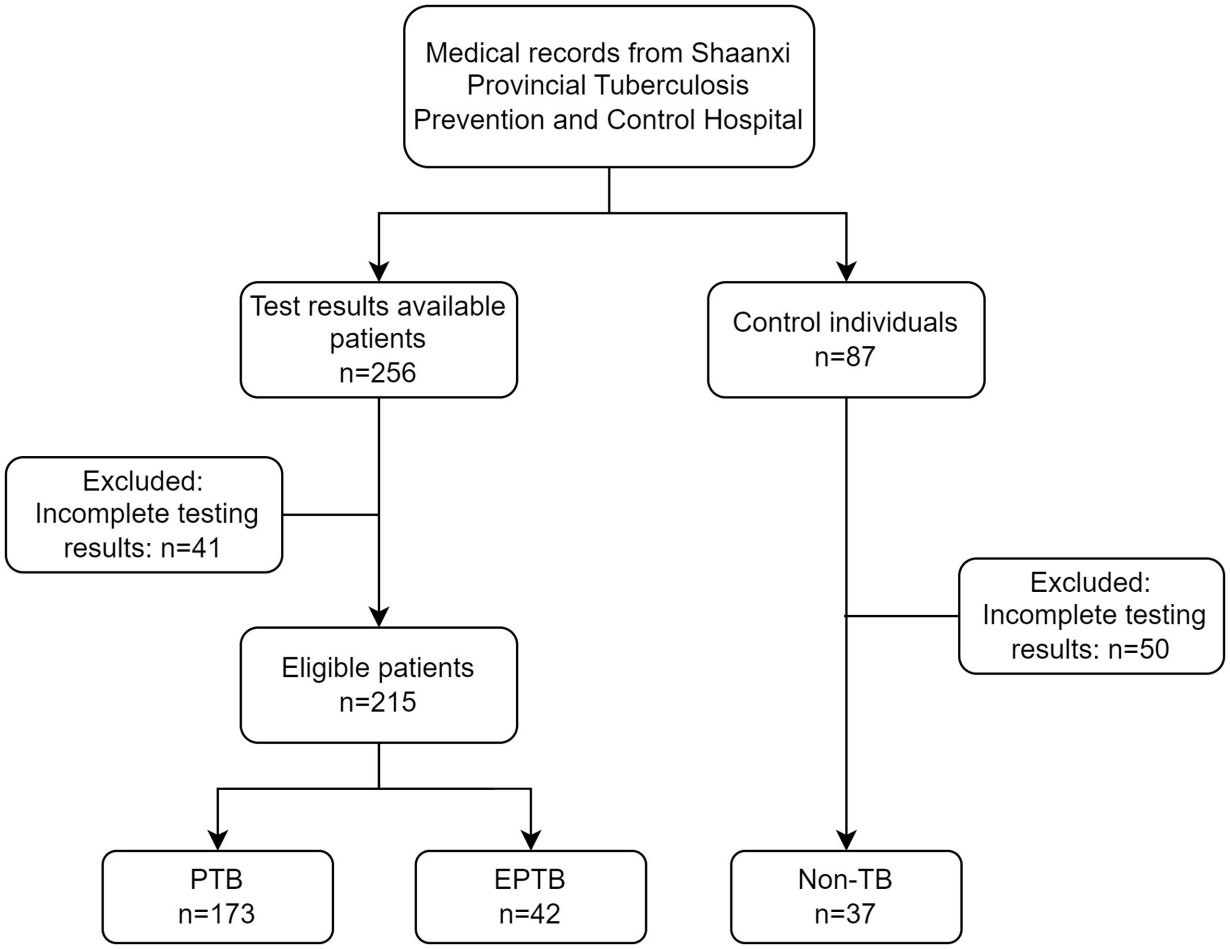
Flow chart of included participants.

This is a retrospectively diagnostic study. 256 patients who were firstly treated during hospitalization in Shaanxi Provincial Tuberculosis Control Hospital from June 2022 to August 2022 were selected for the study. Eligibility criteria were as follows: (1) Patients suffering from pulmonary tuberculosis (PTB) or extra-pulmonary tuberculosis (EPTB) must meet “Diagnosis for pulmonary tuberculosis (WS 288–2017)” (People’s Republic of China state health and Family Planning Commission, 2018) and WHO tuberculosis guideline (World Health Organization, 2022); (2) availability of sputum smears, sputum culture, GeneXpert and urine LAM results. Patients with HIV infection were excluded. 41 cases were excluded from the analysis due to incomplete testing results. The study finally included 215 cases of active tuberculosis patients, including 173 cases of PTB and 42 cases of EPTB. 87 individuals without symptoms related to tuberculosis infection were enrolled from physical examination population in our hospital during the same period, 50 cases were excluded from the analysis due to incomplete testing results. 37 cases were included as the non-TB group (Figure 1). This study adhered to the Helsinki Declaration and was approved by the Medical Ethics Committee of Shaanxi Provincial Tuberculosis Prevention and Control Hospital with the ethics number (2023) LSH-1.

### 2.2 Sample collection

#### 2.2.1 Sputum collection

Sputum was collected from patients during their visit, using sterile physiological saline for mouth rinsing. Sputum was collected in plastic containers or wax-coated paper-sealed containers. Three samples of sputum, collected at night, in the morning, and immediately, were mixed. The volume of each sample was between 2 and 5 mL. The collection was supervised by laboratory personnel. For patients who had difficulty expectorating sputum, sputum induction using hypertonic saline nebulization or bronchoalveolar lavage was employed. The collected sputum specimens were stored at room temperature for up to 2 h before testing or refrigerated at 4°C and were used for testing within 24 h.

#### 2.2.2 Urine collection

Midstream urine was collected to ensure cleanliness, with a minimum volume of 5 mL. After sample collection, it was stored at room temperature and testing was completed within 72 h. The sample should not have undergone more than three freeze–thaw cycles.

#### 2.2.3 Other samples

Other samples mainly included pleural effusion, cerebrospinal fluid, and pus. The puncture was performed under strict aseptic conditions. For pleural effusion, the puncture position was between the 7th and 8th ribs on the scapular line, between the 6th and 7th ribs on the axillary line, or at the 5th rib on the anterior axillary line. For cerebrospinal fluid, the puncture position was between the 3rd and 4th lumbar vertebrae or between the 4th and 5th lumbar vertebrae. First, disinfection was carried out, and then local anesthetics were used for infiltration anesthesia. A puncture needle was used to enter the chest cavity, and pleural effusion was extracted. Finally, the puncture site was covered with sterile dressing.

### 2.3 Laboratory test protocol

#### 2.3.1 Sputum smear

Sputum smear test was conducted using acid-fast stain (AFS) and microscopy. AFB was performed according to the protocol previous reported (Van Deun et al., 2008) followed by microscopical examination. At least 100 visual fields were observed with a “100×” oil microscope, and continued observation of 300 visual fields was performed if no bacilli were found. A negative result was defined as 300 consecutive visual fields with no acid-fast bacilli detected, while the detection of acid-fast bacilli was considered positive.

#### 2.3.2 Sputum culture

Sputum culture was performed according to protocol (Hanna et al., 1999) previously established based on BACTEC™ MGIT ™ 960 TB system (BD Diagnostic Systems, Sparks, MD, USA). NALC+4% NaOH was used to free MTB from the mucus for sputum sample (Wang et al., 2022), tissue specimens were lysed using NALC for tissue lysis. After the pretreatment was completed. Samples were incubated into the BBL MGIT culture tube and then tested. The interpretation of the results was also conducted following the aforementioned protocol.

#### 2.3.3 TB-RNA

TB-RNA testing was performed using simultaneous amplification and testing (SAT) method (Qiu et al., 2022) according to the manufacturer’s instructions (Shanghai Rendu Biological Technology Co., Ltd., China). All samples were diluted with 4% NaOH solution. Diluted sample was centrifuged at 13,000 × g for 5 min then supernatant removed. The resulting pellet was subjected to ultrasound treatment at 300 W for 15 min and then centrifuged to obtain the supernatant. The supernatant was used for the SAT test according to the manufacturer’s instruction.

#### 2.3.4 Urinary LAM detection (chemiluminescence)

Urinary LAM detection was conducted using AIMLAM kits (Leide Biosciences Co., Ltd., China) according to user’s manual (Zhang et al., 2023). This kit uses chemiluminescent immunoassay to detect LAM content in human urine. LAM-captured antibodies on magnetic beads bind to LAM in the test sample, forming a magnetic bead-antibody–antigen complex. After binding with the luminescent label, a magnetic bead-antibody–antigen-aminoluciferin label complex is formed for detection of LAM immune complexes. After separation and washing, the pre-triggering solution and the triggering solution are added to the reaction mixture, and the LAM content in the test substance is proportional to the relative light unit value.

The operating procedure is as follows: 5 mL of midstream urine was collected from the patient and the test was performed according to the instructions. 1.5 mL of the test sample was transferred to a 2 mL centrifuge tube. Then, 50 μL of the magnetic bead reagent (containing LAM-capturing antibodies) was added to the centrifuge tube and mixed. The tube was labeled for identification. The labeled centrifuge tube was placed in a rotating mixer and incubated at room temperature with a rotation speed of 30 rpm/min for 2 h. After incubation, the centrifuge tube was placed on a magnetic rack for adsorption. Once the components were fully separated, the supernatant was discarded. To each tube, 200 μL of sample dilution solution was added. The mixture was thoroughly mixed using a vortex mixer and within 5 min, the sample was processed according to the operation manual of the LAM detection chemiluminescence analyzer SMART 500S (Chongqing Keysmile Biological Technology Co., Ltd., China). If the time exceeded 5 min, the sample needed to be mixed again before testing.

#### 2.3.5 GeneXpert

One mL of sample was collected into a disposable leak-proof 50 mL pre-treatment container. For sputum and lavage fluid samples, a two-fold volume of Sample Reagent (SR) was added (equal volume of SR for body fluid samples). The sample and SR were mixed by shaking for 10–20 times or vortexing for at least 10 s. Subsequently, the mixture was incubated for 15 min at 20–30°C, with another round of mixing at 5–10 min into the incubation period. The processed samples were pipetted into the cartridge using the special sterile pipette provided in the kit, the cartridge was placed in the instrument (Cepheid, USA), and the reading was done by referring to the instructions provided by the manufacturer.

#### 2.3.6 Statistical analysis

The data were analyzed using SPSS 26.0 statistical software (SPSS Inc., Chicago, IL). Qualitative data, such as positive detection rate and sensitivity, were presented as frequencies and percentages (%) and the chi-square test were used. All analyses were two-tailed, and a value of *p* <0.05 was considered statistically significant for differences. According to the composite reference standard, the sensitivity, specificity, positive predictive value (PPV) negative predictive value (NPV), and Kappa value were used to evaluate different methods and consistency. Cost-effectiveness analysis was calculated by dividing the total cost of each diagnostic method by the number of true positive cases diagnosed. The costs were reported in 2022 U.S. dollars, using an exchange rate of 6.74 Chinese yuan to 1 U.S. dollar.^1^

## 3 Results

### 3.1 Demographic data of the participants

A total of 215 patients were included in this study. There were no significant differences in age (*p* = 0.096) and gender (*p* = 0.506) among the PTB, EPTB and Non-TB groups. Regarding comorbidities, there were no significant differences in the prevalence of diabetes (*p* = 0.158), hypertension (*p* = 0.358), coronary heart disease (CHD) (*p* = 0.074), and chronic obstacle pulmonary disease (COPD) (*p* = 0.631). Among PTB patients, 9.25% had a history of tuberculosis (*p* = 0.02), which is higher than that of EPTB patients and Non-TB group (Table 1).

**Table 1:** Demographics of participants.

Variables	All (*N*)	PTB (*N*, %)	EPTB (*N*, %)	Non-TB (*N*, %)	*x* ^2^	*p*
total	252	173 (68.65%)	42 (16.67%)	37 (14.68%)		
gender(male)	131 (51.98%)	90 (52.02%)	17 (40.48%)	24 (64.86%)	4.688	0.096
age					3.319	0.506
<30	56	41 (23.70%)	9 (21.43%)	6 (16.22%)		
30–60	124	83 (47.98%)	24 (57.14%)	17 (45.95%)		
>60	72	49 (28.32%)	9 (21.43%)	14 (37.84%)		
DM	35	28 (16.18%)	2 (4.76%)	5 (13.51%)	3.692	0.158
hypertension	30	24 (13.87%)	3 (7.14%)	3 (8.11%)	2.056	0.358
CHD	20	18 (10.40%)	2 (4.76%)	0 (00.00%)	5.211	0.074
COPD	3	2 (1.16%)	0 (00.00%)	0 (00.00%)	0.921	0.631
history of TB	16	16 (9.25%)	0 (00.00%)	0 (00.00%)	7.802	0.020

PTB, pulmonary tuberculosis; EPTB, extrapulmonary tuberculosis; DM, diabetes mellitus; CHD, chronic heart disease; COPD, chronic obstacle pulmonary disease; TB, tuberculosis.

### 3.2 Diagnostic performance of the five methods compared to CRS

**Figure 2:**
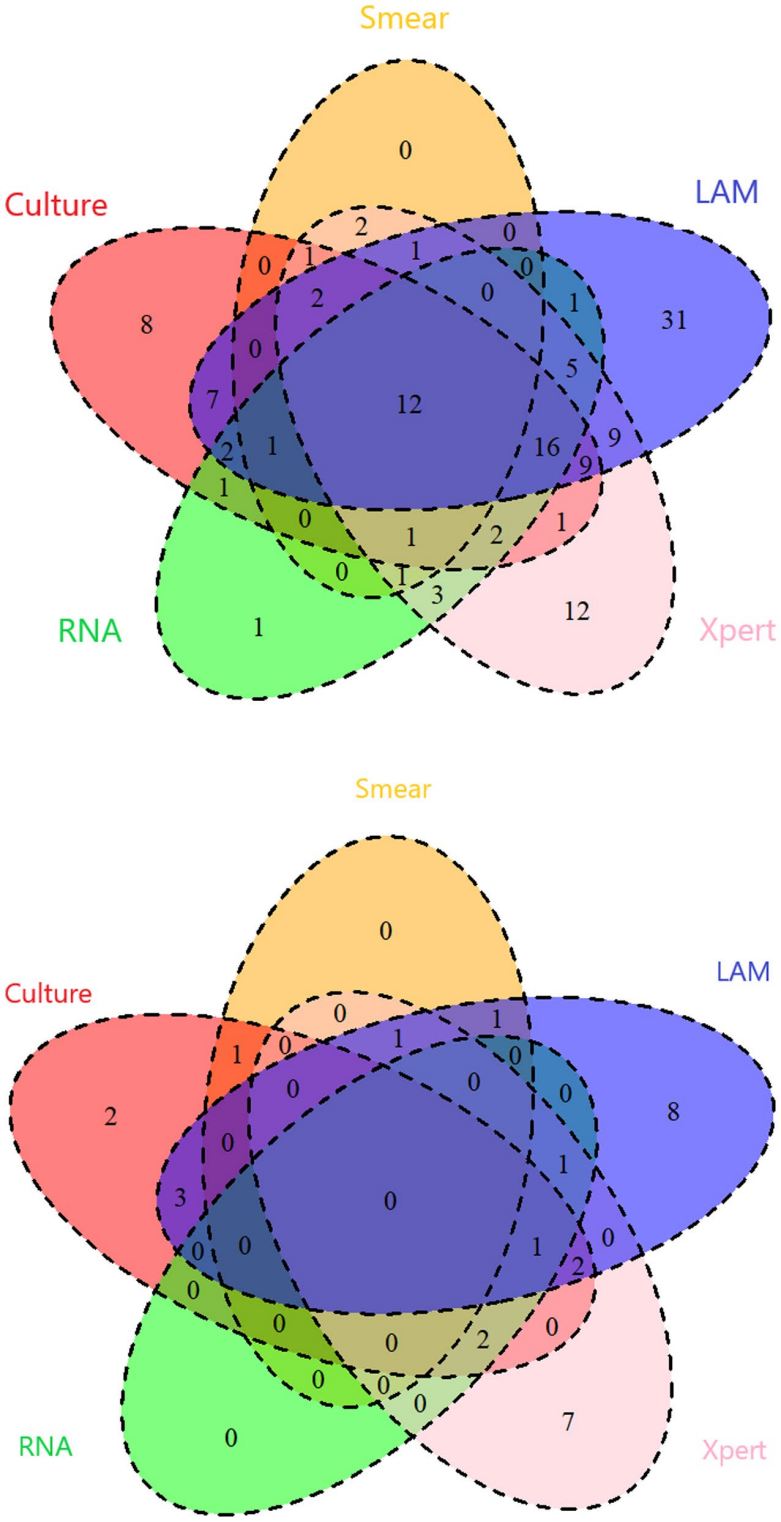
Venn diagram of number of positive individuals of PTB and EPTB.

In PTB, the sensitivity of LAM (55.49%) is significantly higher than that of other methods (*p* < 0.05). The specificity and PPV of all five methods reached 100%. LAM demonstrates the highest NPV (32.46%), slightly higher than Xpert (27.82%). The kappa value for LAM is also the highest (0.305), higher than Xpert (0.220) (Table 2). In EPTB, LAM shows the highest sensitivity, NPV, and kappa value (40.48, 59.68%, 0.389, respectively), followed by GeneXpert (33.33, 56.92%, 0.319, respectively). The sensitivity of LAM is higher than smear (7.14%, *p* < 0.05) and RNA (9.52%, *p* < 0.05). The specificity and PPV for all five methods were 100% (Table 3). Positive results of PTB and EPTB patients’ tests are shown in Figure 2.

**Table 2:** The diagnostic performance of five methods in PTB.

Methods	Results	CRS		Sensitivity	Specificity	PPV	NPV	Kappa
		PTB	Non-TB					
smear	positive	21	0	12.14%^*^	100.00%	100.00%	19.58%	0.046
	negative	152	37					
culture	positive	63	0	36.42%^*^	100.00%	100.00%	25.17%	0.168
	negative	110	36					
RNA	positive	46	0	26.59%^*^	100.00%	100.00%	22.56%	0.113
	negative	127	37					
Xpert	positive	77	0	44.51%^*^	100.00%	100.00%	27.82%	0.220
	negative	96	37					
LAM	positive	96	0	55.49%	100.00%	100.00%	32.46%	0.305
	negative	77	37					

CRS, composite reference standard; PTB, pulmonary tuberculosis; PPV, positive predictive value; NPV, negative predictive value; RNA, ribonucleic acid; TB: tuberculosis; LAM, lipoarabinomannan; *p < 0.05, Compared to LAM.

**Table 3:** The diagnostic performance of five methods in EPTB.

Methods	Results	CRS		Sensitivity	Specificity	PPV	NPV	Kappa
		EPTB	Non-TB					
smear	positive	3	0	7.14%^*^	100.00%	100.00%	48.68%	0.067
	negative	39	37					
culture	positive	11	0	26.19%	100.00%	100.00%	54.41%	0.249
	negative	31	37					
RNA	positive	4	0	9.52%^*^	100.00%	100.00%	49.33%	0.090
	negative	38	37					
Xpert	positive	14	0	33.33%	100.00%	100.00%	56.92%	0.319
	negative	28	37					
LAM	positive	17	0	40.48%	100.00%	100.00%	59.68%	0.389
	negative	25	37					

CRS, composite reference standard; EPTB, extrapulmonary tuberculosis; PPV, positive predictive value; NPV, negative predictive value; RNA, ribonucleic acid; TB, tuberculosis; LAM, lipoarabinomannan; *p < 0.05, Compared to LAM.

### 3.3 Diagnostic performance of LAM in combination of other methods

In PTB, LAM combined with another diagnostic method showed the highest sensitivity when combined with GeneXpert (68.79%), followed by combination with sputum culture (63.58%), RNA (60.69%), and sputum smear (58.38%). The sensitivity of LAM in combination with the other four methods was significantly higher than when they were individually tested (*p* < 0.05). The sensitivity of LAM in combination with Xpert was higher than when LAM was tested alone (*p* < 0.05). Compared to individual detection methods, LAM detection in combination with sputum smear, sputum culture, RNA, and GeneXpert showed higher sensitivity, increasing by 46.24, 27.17, 34.10 and 24.28%, respectively, (Table 4). When LAM, Xpert were combined with another methods for diagnosing PTB, the combination of culture, Xpert, and LAM showed the highest sensitivity (73.99%) (Table 5). The sensitivity of all three combination approaches was higher than that of using LAM alone (*p* < 0.05). In EPTB, the combined use of LAM with other diagnostic methods can significantly improve sensitivity. According to the data provided, the combination of LAM with GeneXpert showed the highest sensitivity (61.90%), followed by the combination of LAM with sputum culture (52.38%), RNA (45.24%), and sputum smear (42.86%). The sensitivity of LAM in combination with the other four methods was significantly higher than when they were individually tested (*p* < 0.05). The sensitivity of LAM in combination with Xpert was higher than when LAM was tested alone (*p* < 0.05). Compared to individual detection methods, the combination of LAM with sputum smear, sputum culture, RNA, and GeneXpert can increase sensitivity by 35.71, 26.19, 35.71, and 28.57%, respectively (Table 6). When LAM is combined with two other methods (culture and Xpert) for the diagnosis of EPTB, the sensitivity is highest at 69.05% (Table 7). The sensitivity of all three combination approaches was higher than that of using LAM alone (*p* < 0.05).

**Table 4:** Diagnostic performance of LAM in combination of 1 other method in PTB.

Methods	Results	CRS		Sensitivity	Increment	Specificity	PPV	NPV	Kappa
		PTB	Non-TB						
LAM + smear	positive	101	0	58.38%^#^	46.24%	100.00%	100.00%	33.94%	0.331
	negative	72	37						
LAM + culture	positive	110	0	63.58%^#^	27.17%	100.00%	100.00%	37.00%	0.381
	negative	63	37						
LAM + RNA	positive	105	0	60.69%^#^	34.10%	100.00%	100.00%	35.24%	0.352
	negative	68	37						
LAM + Xpert	positive	119	0	68.79%*^#^	24.28%	100.00%	100.00%	40.66%	0.437
	negative	54	37						

CRS, composite reference standard; PTB, pulmonary tuberculosis; PPV, positive predictive value; NPV, negative predictive value; RNA, ribonucleic acid; TB, tuberculosis; LAM, lipoarabinomannan; *p < 0.05, Compared to LAM; ^#^*p* < 0.05, compared to single detection.

**Table 5:** Diagnostic performance of LAM in combination of 2 other methods in PTB.

Methods	Results	CRS		Sensitivity	Increment	Specificity	PPV	NPV	Kappa
		PTB	Non-TB						
LAM + Xpert+smear	positive	119	0	68.79%*	13.29%	100.00%	100.00%	40.66%	0.437
	negative	54	37						
LAM + Xpert+culture	positive	128	0	73.99%*	18.50%	100.00%	100.00%	45.12%	0.501
	negative	45	37						
LAM + Xpert+RNA	positive	121	0	69.94%*	14.45%	100.00%	100.00%	41.57%	0.451
	negative	52	37						

CRS, composite reference standard; PTB, pulmonary tuberculosis; PPV, positive predictive value; NPV, negative predictive value; RNA, ribonucleic acid; TB, tuberculosis; LAM, lipoarabinomannan; *p < 0.05, Compared to LAM.

**Table 6:** Diagnostic performance of LAM in combination of 1 other method in EPTB.

Methods	Results	CRS		Sensitivity	Increment	Specificity	PPV	NPV	Kappa
		EPTB	Non-TB						
LAM + smear	positive	18	0	42.86%^#^	35.71%	100.00%	100%	60.66%	0.413
	negative	24	37						
LAM + culture	positive	22	0	52.38%^#^	26.19%	100.00%	100%	64.91%	0.507
	negative	20	37						
LAM + RNA	positive	19	0	45.24%^#^	35.71%	100.00%	100%	61.67%	0.436
	negative	23	37						
LAM + Xpert	positive	26	0	61.90%*^#^	28.57%	100.00%	100%	69.81%	0.604
	negative	16	37						

CRS, composite reference standard; EPTB, extrapulmonary tuberculosis; PPV, positive predictive value; NPV, negative predictive value; RNA, ribonucleic acid; TB, tuberculosis; LAM, lipoarabinomannan; *p < 0.05, Compared to LAM; ^#^*p* < 0.05, compared to single detection.

**Table 7:** Diagnostic performance of LAM in combination of 2 other method in EPTB.

Methods	Results	CRS		Sensitivity	Specificity	PPV	NPV	Kappa
		EPTB	Non-TB					
LAM + Xpert+RNA	positive	26	0	61.90%*	100.00%	100.00%	69.81%	0.604
	negative	16	37					
LAM + Xpert+culture	positive	29	0	69.05%*	100.00%	100.00%	74.00%	0.676
	negative	13	37					
LAM + Xpert +smear	positive	27	0	64.29%*	100.00%	100.00%	71.15%	0.628
	negative	15	37					

CRS, composite reference standard; EPTB, extrapulmonary tuberculosis; PPV, positive predictive value; NPV, negative predictive value; RNA, ribonucleic acid; TB, tuberculosis; LAM, lipoarabinomannan; *p < 0.05, Compared to LAM.

### 3.4 Cost-effective analysis of different combination

The calculation method for cost-effectiveness is to divide the total cost (unit price multiplied by the number of participants) by the number of true positive cases. An analysis of the economic benefits of the aforementioned combination approaches reveals that when used alone for the diagnosis of PTB, the *per capita* cost of LAM is lower than Xpert ($129.82 vs. $242.78) but higher than other methods. When using the lowest national price, LAM is priced lower than Xpert and RNA, but higher than others. When using the highest national price, LAM is priced lower than Xpert but higher than others. For EPTB, the *per capita* cost of LAM is lower than RNA and Xpert ($275.79, $468.84 and $502.33respectively), but still higher than other methods. The same applies when using the highest and lowest national prices. When combined with another method for PTB diagnosis, the combination of LAM and smear has the lowest cost ($124.94). The same applies when using the highest and lowest national prices. Similarly, for EPTB, the combination of LAM and smear also has the lowest cost ($263.72). The same applies when using the highest and lowest national prices. When combined with Xpert and another method for PTB diagnosis, the combination of Xpert, RNA, and LAM has the lowest cost ($258.78). The combination of Xpert, smear, and LAM has the lowest cost ($436.28) in EPTB diagnosis. When using the highest and lowest national prices, Xpert, smear, and LAM has the lowest cost (Table 8).

**Table 8:** The cost-effectiveness analysis [$, price in Shaanxi (lowest nationally, highest nationally)].

Methods	Price	PTB	EPTB
smear	0.74 (0.74,2.23)	7.42 (7.42,22.26)	19.54 (19.54,58.61)
culture	20.77 (9.29,29.67)	69.24 (30.96,98.91)	149.18 (66.7,213.11)
RNA	23.74 (11.19,23.74)	108.37 (51.07,108.37)	468.84 (220.94,468.84)
Xpert	89.02 (69.44,90.5)	242.78 (189.37,246.83)	502.33 (391.82,510.7)
LAM	59.35 (22.26,59.35)	129.82 (48.68,129.82)	275.79 (103.42,275.79)
LAM+ smear	60.09 (23,61.57)	124.94 (47.82,128.02)	263.72 (100.93,270.24)
LAM+ culture	80.12 (31.54,89.02)	152.95 (60.22,169.95)	287.7 (113.27,319.67)
LAM+ RNA	83.09 (33.44,83.09)	166.17 (66.88,166.17)	345.46 (139.05,345.46)
LAM+ Xpert	148.37 (91.69,149.85)	261.83 (161.81,264.44)	450.81 (278.6,455.32)
LAM + Xpert+RNA	172.11 (102.88,173.59)	258.78 (178.55,301.27)	453.06 (312.59,527.45)
LAM + Xpert+culture	169.14 (100.98,179.53)	277.49 (165.67,294.53)	460.76 (275.08,489.05)
LAM + Xpert +smear	149.11 (92.43,152.08)	263.14 (163.12,268.37)	436.28 (270.45,444.97)

PTB, pulmonary tuberculosis; EPTB, extrapulmonary tuberculosis; RNA, ribonucleic acid; LAM, lipoarabinomannan.

## 4 Discussion

This study investigated the diagnostic value of LAM, sputum culture, sputum smear, RNA, and GeneXpert for the diagnosis of PTB and EPTB. We also compared the diagnostic efficacy of LAM in combination with other diagnostic methods. The main finding of this study is that chemiluminescent-based LAM achieved a sensitivity of 55.49% in PTB and 40.48% in EPTB (Tables 2, 3), Which is higher than the sensitivity of other diagnostic methods, in this study, the sensitivity of sputum smear microscopy is 12.14%, sputum culture approximately 36.42%, and GeneXpert 44.51%, exhibiting relatively lower levels when compared to other institutions (Sorsa and Kaso, 2021). This difference in sensitivity might be attributed to variances in sample collection and testing procedures among individual laboratories.

Moreover, a meta-analysis reported that the sensitivity of LAM detection for PTB in HIV-negative populations was only about 31% (Yin et al., 2022). The currently available commercial LAM detection method, Alere’s Determine TB LAM, has limitations in methodology, resulting in lower sensitivity in HIV-negative populations. Therefore, it is not suitable for TB diagnosis in HIV-negative individuals (Suwanpimolkul et al., 2017). Our study significantly improves the sensitivity of LAM detection in PTB or EPTB. To enhance the sensitivity of LAM detection, raising the detection limit is crucial. Chemiluminescence has a high signal-to-noise ratio and is one of the methods with the highest analytical sample concentration (Green et al., 2017). Additionally, the use of high-affinity monoclonal antibodies and magnetic bead concentration technology in the kit significantly enhances the detection limit of LAM (Peng et al., 2023). Our findings exhibit a similar sensitivity and specificity compared to those reported in prior studies employing the same LAM testing method (Huang et al., 2023; Zhang et al., 2023).

In clinical practice, multiple diagnostic methods are often used in combination to improve the detection rate of TB patients. Therefore, we conducted a comparative analysis of the diagnostic performance of combining LAM with other methods for TB diagnosis. When combined with another method for TB diagnosis, the sensitivity of several other methods was significantly improved. In PTB (Table 4), the combination of LAM with smear increased the sensitivity by over 23 to 40% compared to using smear alone, and the sensitivity of combinations with four other methods exceeded 50%. The combination of LAM with Xpert showed the highest sensitivity (68.79%). The research results of Shah et al. also showed that the sensitivity of combined detection using Xpert and LAM was superior to using either detection method alone (Shah et al., 2014). The result is similar to Zhang et al. (2023). In EPTB (Table 6), the sensitivity was increased by 26 to 36%, and again, the combination of LAM with Xpert showed the highest sensitivity (61.90%). When combined with two other diagnostic methods for pulmonary TB (Table 5), the combination of culture, Xpert, and LAM exhibited the highest sensitivity at 73.99%. Similarly, in EPTB (Table 7), the combination of culture, Xpert, and LAM also demonstrated the highest sensitivity at 69.05%. From the perspective of improving sensitivity, the combination of LAM, Xpert, and culture is the optimal approach for both EPTB and PTB patients.

The incidence of tuberculosis is positively correlated with the economic level of countries and regions, with higher rates observed in economically disadvantaged areas. According to the Global Tuberculosis Report of 2023, geographically, the regions with the highest number of tuberculosis patients in 2022 were the WHO Southeast Asia region (46%), Africa (23%), and the Western Pacific region (18%), primarily concentrated in areas with poor economic development (World Health Organization, 2023). In western China, a region characterized by economic underdevelopment, the burden of tuberculosis is particularly heavy (Hu et al., 2022). Therefore, reducing the cost-effectiveness ratio of tuberculosis diagnosis is essential for ending the tuberculosis epidemic.

The significant cost disparity among various diagnostic techniques, with a nominal expense of $0.74 for sputum smear compared to the higher cost of $89.02 for GeneXpert, underscores the need for judicious selection of diagnostic methods, especially in resource-constrained settings and screening initiatives. Taking into account the price variations for each diagnostic test across different regions in the country, we conducted a cost-effectiveness analysis using pricing standards specific to Shaanxi Province, the highest national pricing, and the lowest national pricing.

To address this, we conducted a comprehensive cost-effectiveness analysis encompassing diverse diagnostic methods and combinations for both PTB and EPTB. Sputum smear emerges as the most economically viable single detection method, with costs amounting to $7.42 for PTB and $19.54 for EPTB, respectively. Among combinations exhibiting sensitivity surpassing 50%, the most cost-effective option is the combination of sputum smear with LAM, incurring expenses of $124.94 for PTB and $263.72 for EPTB. These results hold consistent when considering both the lowest and highest national pricing standards. Given the shorter time requirements for LAM detection and smear microscopy, these methods play a crucial role in expediting the diagnosis of tuberculosis patients. Furthermore, if combined with sputum culture for diagnosis, a marginal cost increase is observed, significantly augmenting diagnostic capabilities. When the price of LAM is as low as $22.26 or even lower, its cost-effectiveness for diagnosing PTB ($48.68) and EPTB ($103.42) is only secondary to sputum smear and sputum culture. This further emphasizes the importance of LAM, especially in resource-limited settings. By using LAM in combination with sputum smear, diagnostic accuracy can be significantly improved while saving time and costs during the diagnosis process. Furthermore, although it may slightly increase the cost, combining with sputum culture for diagnosis can significantly enhance diagnostic capabilities. Therefore, when formulating diagnostic strategies and allocating resources, the pricing of LAM and cost-effectiveness ratios of other diagnostic methods should be taken into account to ensure optimal diagnostic outcomes in resource-limited settings.

This study has the following limitations: On the one hand, it is a single-center retrospective study, which is subject to inherent biases associated with retrospective studies. Future research could be conducted as multi-center and prospective studies. On the other hand, given the complexity of medical activities, such as the different operation and interpretation times required for different diagnostic methods, our cost-effectiveness analysis did not take these factors into account. Future research can use more comprehensive methods, such as decision tree models, to analyze the cost-effectiveness of different diagnostic methods.

## 5 Conclusion

Urine LAM detection based on chemiluminescence assay can be used as an adjunct diagnostic tool for PTB and EPTB in HIV-negative individuals. This facilitates expanding the current application of urine LAM from solely HIV-positive populations to the general population. LAM detection can overcome the limitations of obtaining clinical samples, and its ease of sample acquisition will be beneficial for its broader application in a larger scope. For economically better-off areas, we recommend using a combination of LAM + Xpert+culture for higher sensitivity; for economically disadvantaged areas, LAM + smear microscopy combination can provide a quick and accurate diagnosis of tuberculosis at a lower cost.

## Data availability statement

The raw data supporting the conclusions of this article will be made available by the authors, without undue reservation.

## Ethics statement

The studies involving humans were approved by Ethics Committee, Shaanxi Provincial Tuberculosis Prevention and Control Hospital. The studies were conducted in accordance with the local legislation and institutional requirements. The human samples used in this study were acquired from a by-product of routine care or industry. Written informed consent for participation was not required from the participants or the participants’ legal guardians/next of kin in accordance with the national legislation and institutional requirements.

## Author contributions

MG: Conceptualization, Formal analysis, Writing – original draft. QW: Investigation, Methodology, Writing – review & editing. XW: Data curation, Writing – review & editing. XS: Validation, Visualization, Writing – review & editing. ML: Software, Writing – review & editing. GB: Conceptualization, Project administration, Writing – review & editing.

## Footnotes

^1^ https://wdi.worldbank.org/table/4.16
